# Supplementary material for: Systematic evaluation of blood contamination in nanoparticle-based plasma proteomics
Source: EMBO Mol Med. 2025 Dec 5;18(1):275–96. doi: 10.1038/s44321-025-00346-9 (PMC12808129; doi:10.1038/s44321-025-00346-9)
Supplement: Supplementary file 1 — Table EV1 [file 44321_2025_346_MOESM1_ESM.docx]

Table EV1

| **Age** | **Height (cm)** | **Weight (kg)** | **Sex** | **Smorking history** | **CEA (ng/mL)** | **CYFRA 21-1 (ng/mL)** | **SCCA** **(ng/mL)** | **NSE (ng/mL)** | **CA-125 (U/mL)** | **CA 19-9 (U/mL)** | **Nodule size** | **Staging** | **Medical history** | **Medication history** |
| --- | --- | --- | --- | --- | --- | --- | --- | --- | --- | --- | --- | --- | --- | --- |
| 62 | 151 | 52.7 | M | NO | 1.3 | 1.1 | 0.7 | 13.1 | 13.2 | 4.1 | / | / | Benign | Hypertension, Diabetes mellitus |
| 70 | 172 | 68 | M | NO | 3 | 1.1 | 1.4 | 22.5 | 28.5 | 14.1 | Mass, Ground-glass nodule | 41×36 mm, 11×10 mm | IA2 | / |
| 62 | 168 | 68 | F | NO | 2.9 | 2.1 | 0.8 | 14.7 | 11.3 | 12.2 | / | / | ⅠA3 | / |
| 73 | 158 | 60 | M | NO | - | - | - | - | - | - | Part-solid nodule | 28×17 mm | Benign | / |
| 61 | 152 | 49 | F | NO | 1.6 | 1.9 | 0.9 | 23.1 | - | 3.9 | Part-solid nodule | 5×5 mm | IA1 | / |
| 73 | 169 | 71.5 | M | NO | 1.6 | 2.2 | 1.9 | 18.3 | 9.9 | 7.9 | Ground-glass nodule | 13×12mm, 11×16 mm | IA1 | / |
| 32 | 156 | 57.5 | F | NO | - | 0.4 | 0.9 | 11.1 | - | - | Solid nodule | 8×6 mm, 4×3 mm | IA1 | / |
| 53 | 163 | 65 | F | NO | 1 | 1 | 1.4 | 16.3 | 7.4 | 12.3 | Ground-glass nodule | 6×5 | IA1 | / |
| 57 | 170 | 70 | M | NO | 1.9 | 1.2 | 0.9 | 12.8 | 10.1 | 2.6 | Ground-glass nodule, Solid nodule | 18×15 mm, 5×4 mm | IA3 | / |
| 47 | 177.5 | 80 | F | NO | 2.6 | 0.9 | 0.6 | 14.7 | 6.8 | 2.4 | Part-solid nodule, Ground-glass nodule | 9×7 mm, 3×3 mm | Benign | / |

**Table EV1 - Patient information for plasma samples used in the blood collection tube comparison study.**
