# Supplementary material for: Systematic evaluation of blood contamination in nanoparticle-based plasma proteomics
Source: EMBO Mol Med. 2025 Dec 5;18(1):275–96. doi: 10.1038/s44321-025-00346-9 (PMC12808129; doi:10.1038/s44321-025-00346-9)
Supplement: Supplementary file 2 — Appendix [file 44321_2025_346_MOESM2_ESM.pdf]

Appendix Figures

Figure	Title	Page
Appendix Figure S1	Comparative analysis of various SiO_2-based nanoparticles	2
Appendix Figure S2	The optimization of parameters influencing protein corona formation	3
Appendix Figure S3	Nanoparticle concentration effects on protein corona formation and flocculation	4
Appendix Figure S4	Relationship between coefficient of variation (CV) and protein abundance across identified proteins	5
Appendix Figure S5	Identified peptides and protein groups in various plasma samples with varying platelet contamination levels	6
Appendix Figure S6	Correlation analysis of platelet and erythrocyte markers from Geyer's study in neat and OmniProt plasma samples	7
Appendix Figure S7	Correlation of platelet-related markers from Geyer's study in plasma samples prepared using the neat and OmniProt workflows	8
Appendix Figure S8	Proteome analysis across varying degrees of platelet contamination levels. Number of identified peptide precursors	9
Appendix Figure S9	Comparative analysis of protein abundance distributions in matched PRP and PPP samples	10
Appendix Figure S10	Heatmap of protein identification in 11 paired PRP and PPP samples	11
Appendix Figure S11	Comparison of platelet-rich plasma (PRP) samples and platelet-poor plasma (PPP) samples obtained using various centrifugation methods	12
Appendix Figure S12	Erythrocyte contamination effects on OmniProt plasma proteome analysis	13
Appendix Figure S13	Correlation analysis of erythrocyte markers in OmniProt plasma samples	14
Appendix Figure S14	Validation of 30 erythrocyte markers in contaminated plasma samples	15

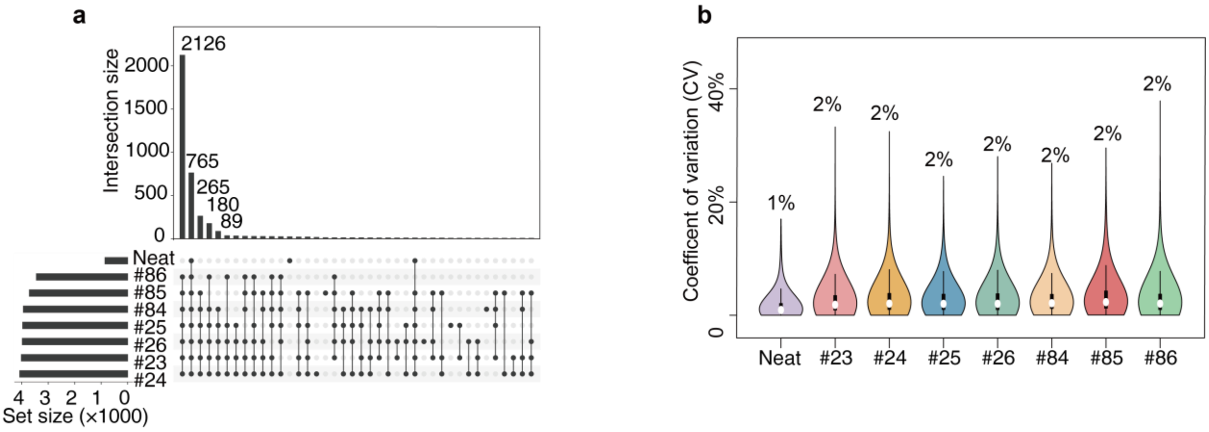

Appendix Figure S1 - **Comparative analysis of various SiO_2-based nanoparticles.**

(a) Upset diagram of identified protein groups across various SiO_2-based nanoparticles. (b) Coefficients of variation for identified protein groups across various SiO_2-based nanoparticles. The numbers above each violin plot in panel (b) represent the median coefficient of variation across biological triplicates.

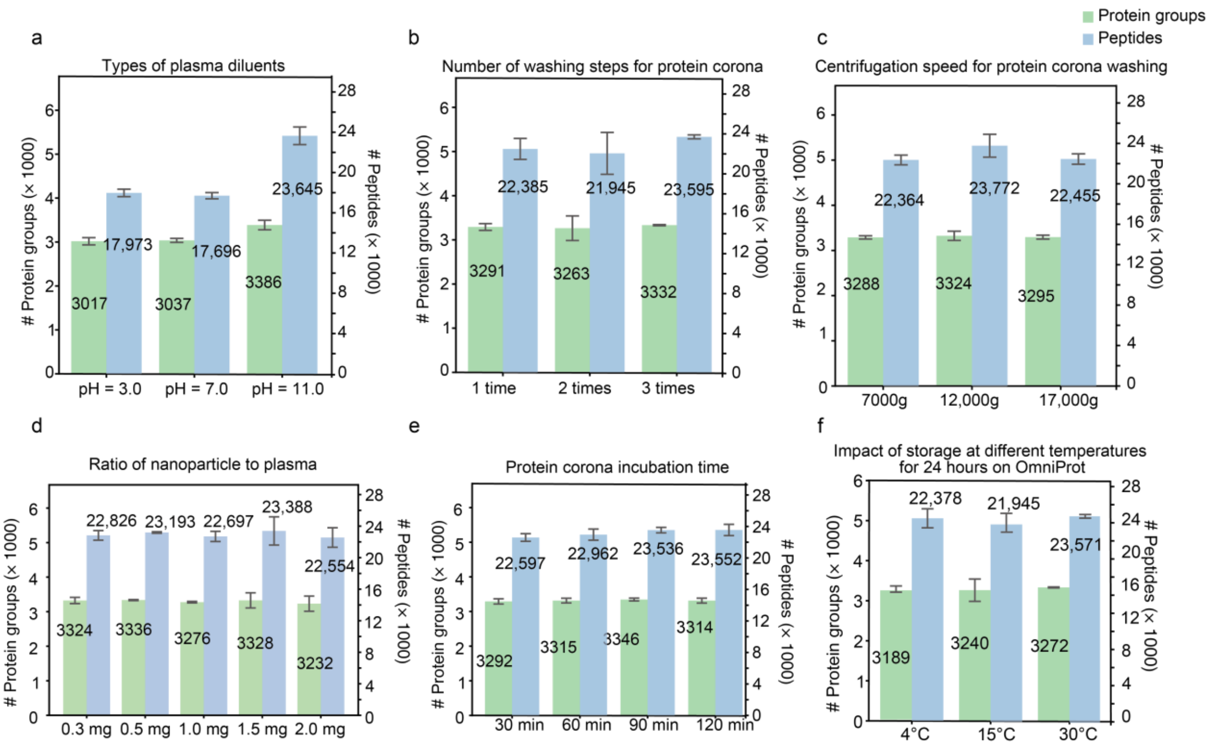

Appendix Figure S2 - **The optimization of parameters influencing protein corona formation.** Parameters influencing protein corona formation included types of plasma diluents (a), number of washing steps for protein corona (b), centrifugation speed for protein corona washing (c), ratio of nanoparticle to plasma (d), protein corona incubation time (e), and impact of 24-hours storage at different temperatures (f) on protein identification. Data represent mean ± standard error of the mean from biological triplicates.

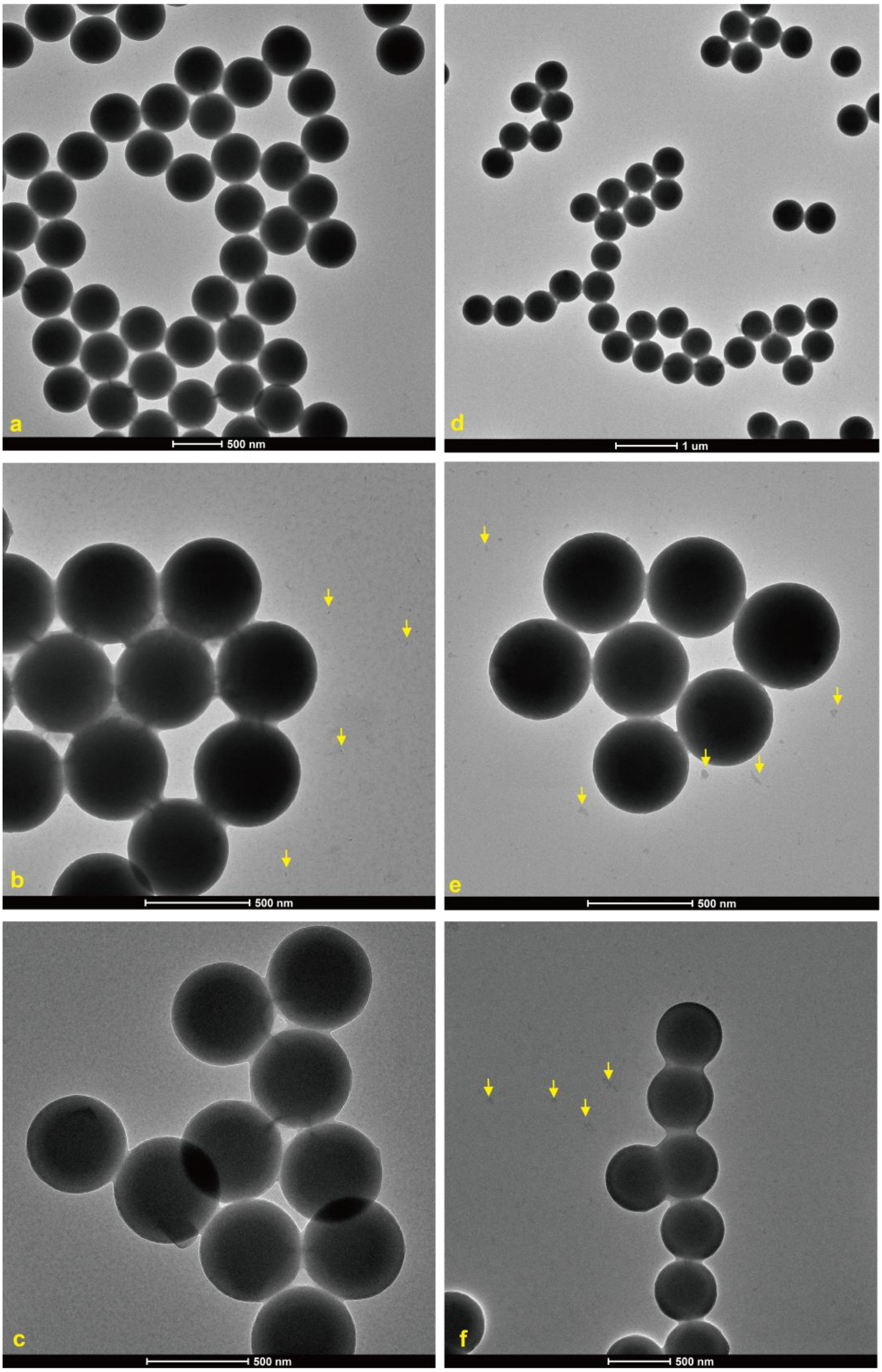

Appendix Figure S3 - **Nanoparticle concentration effects on protein corona formation and flocculation.**

(a-c) NP23 at 2.78 mg/mL reaction concentration. (a) Low-magnification RT-TEM, (b) high-magnification RT-TEM, (c) high-magnification Cryo-TEM. (d-f) NP23 at 5.55 mg/mL reaction concentration. (d) Low-magnification RT-TEM, (e) high-magnification RT-TEM, (f) high-magnification Cryo-TEM. RT-TEM: room temperature transmission electron microscopy. Cryo-TEM: Cryo-transmission electron microscopy.

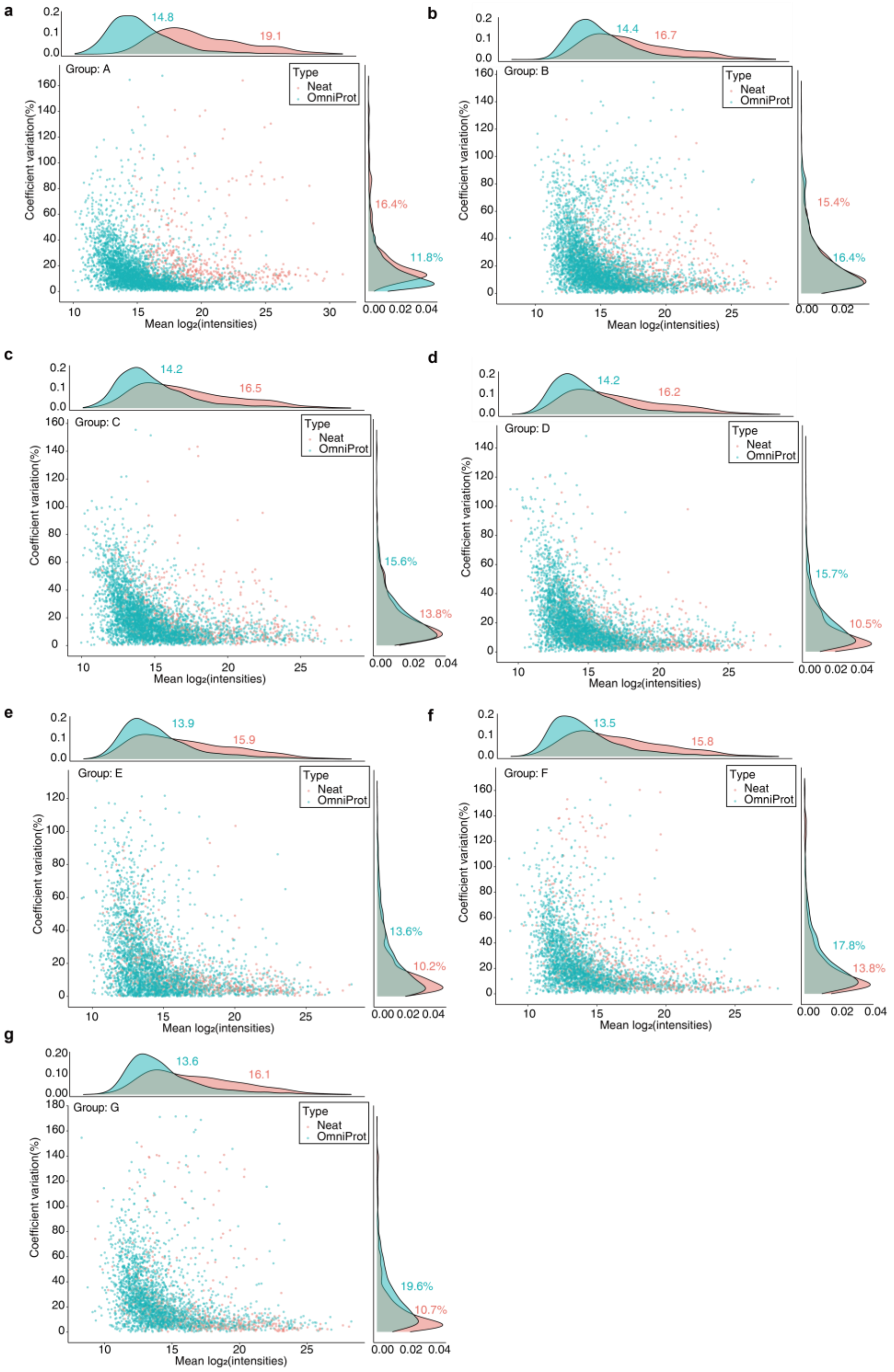

Appendix Figure S4 - **Relationship between coefficient of variation (CV) and protein abundance across identified proteins.**

(a-g) Coefficient of variation plotted against protein abundance for bovine-human mixture groups A-G (as defined in **Figure 3a**). Each dot represents an individual protein with CV calculated from biological triplicate analyses.

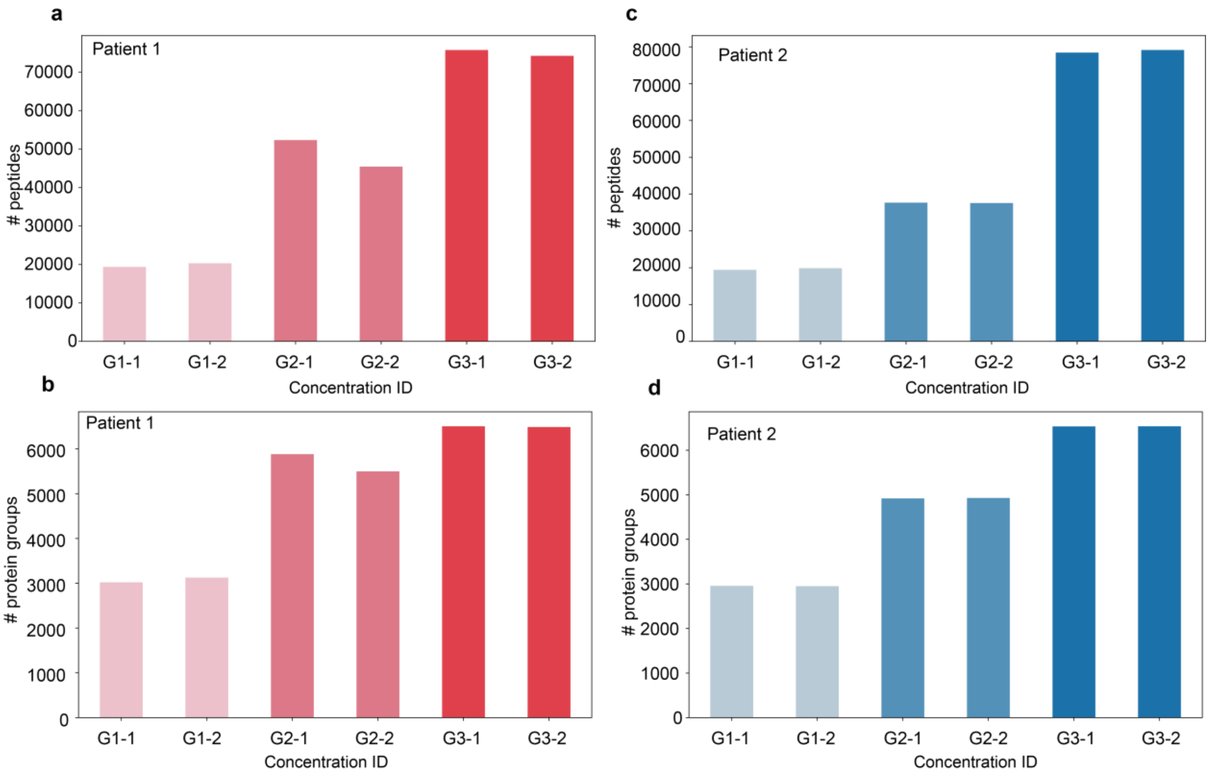

Appendix Figure S5 - **Identified peptides and protein groups in plasma samples with varying platelet contamination levels.**

Number of identified peptides (a, c) and protein groups (b, d) in plasma samples from patients 1 and 2, respectively. G1, G2, and G3 represent low, moderate, and high platelet contamination levels, with biological duplicates (1 and 2) for each group.

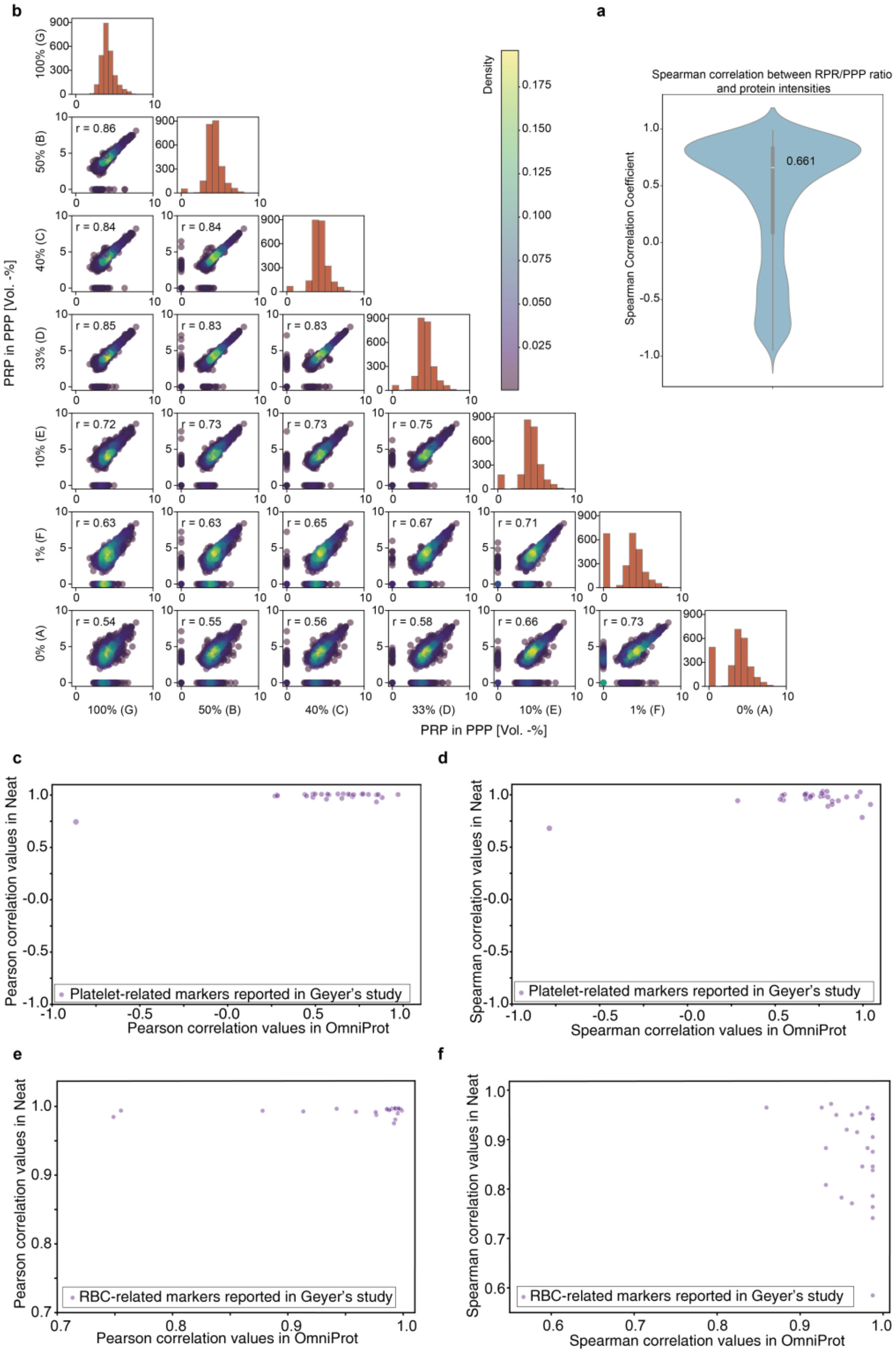

Appendix Figure S6 - **Correlation analysis of platelet and erythrocyte markers from Geyer's study in neat and OmniProt plasma samples.**

(a) Spearman correlation distribution between RPR/PPP ratio and protein intensities. (b) Pearson correlation analysis of the 2432 platelet-independent proteins across samples A-G with varied PRP-to-PPP ratios, as described in **Figure 4a**. (c) Pearson and (d) Spearman correlations of platelet-related markers between neat and OmniProt samples. (e) Pearson and (f) Spearman correlations of RBC-related markers between neat and OmniProt samples.

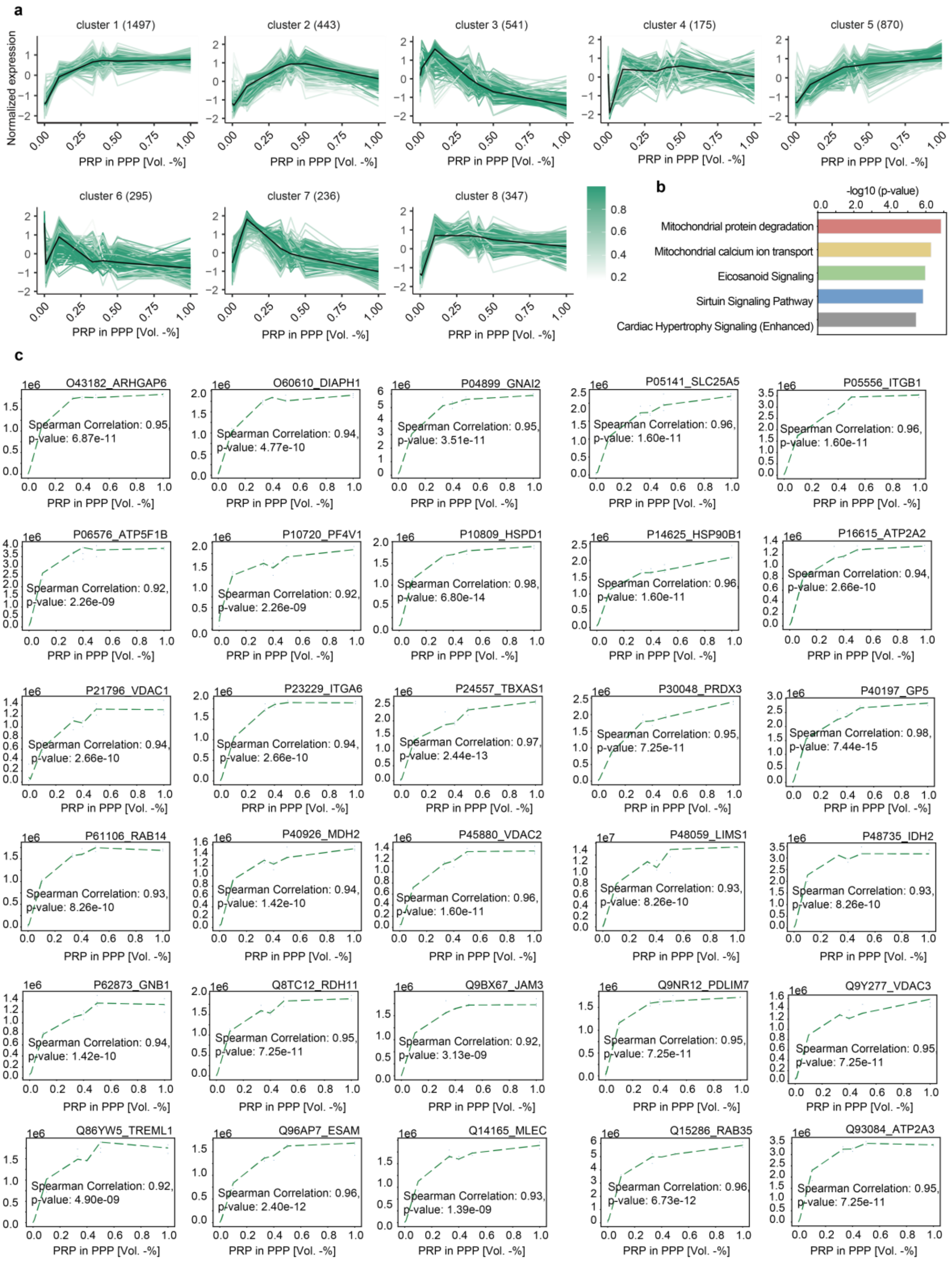

Appendix Figure S7 - **Correlation of platelet-related markers from Geyer's study in plasma samples prepared using the neat and OmniProt workflows.**

(a) Mfuzz clustering analysis of proteins with <50% missing values. (b) Pathway enrichment analysis of identified 30 platelet-related markers. (c) Spearman correlation for 30 markers in platelet discovery dataset.

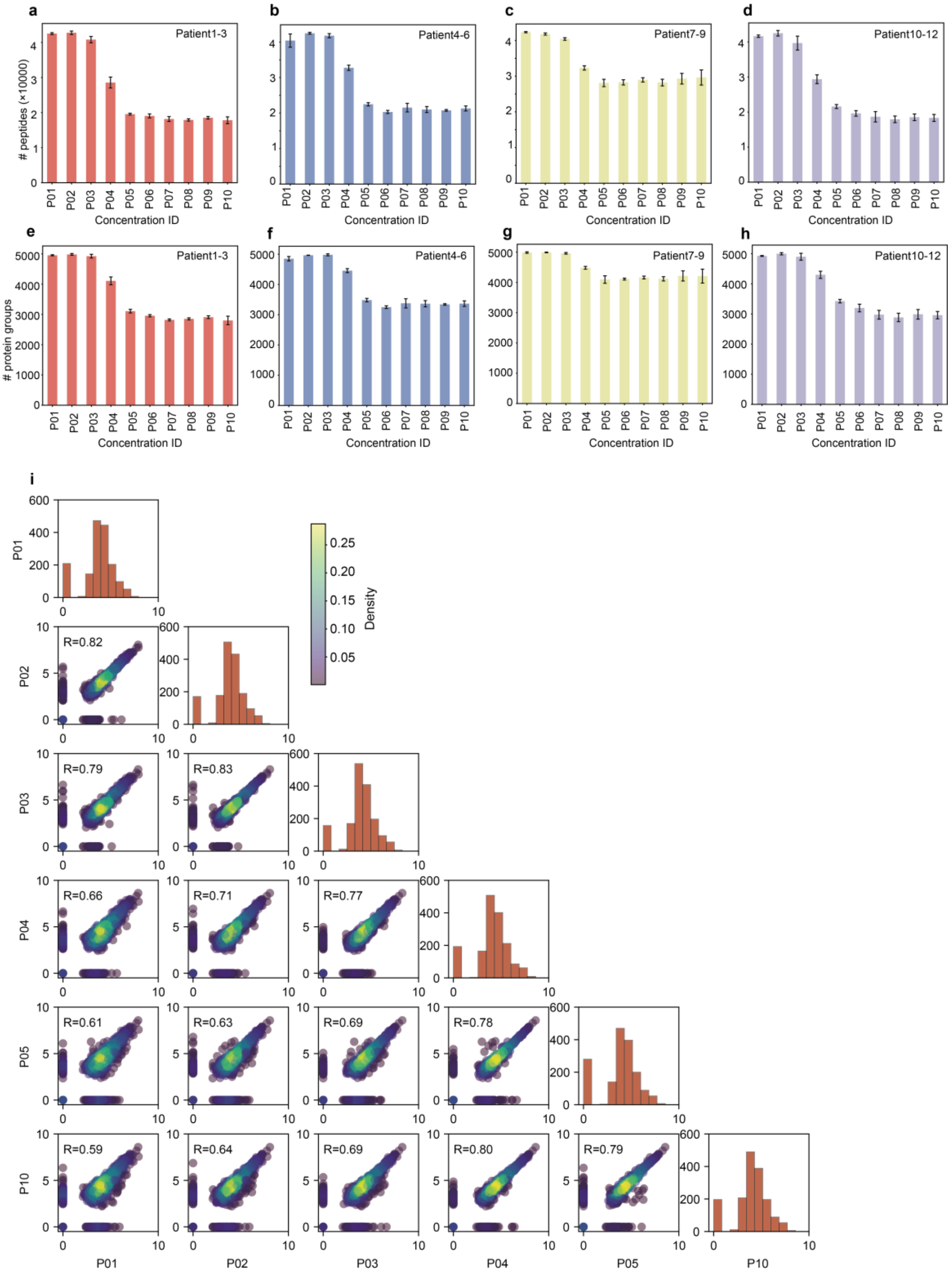

Appendix Figure S8 - **Proteome analysis across varying degrees of platelet contamination levels.** Number of identified peptide precursors (a-d) and protein groups (e-h) in 10 samples (P01-P10) with different platelet contamination levels from four pooled patient samples (n = 12). (i) Pearson correlation analysis of plasma proteomes across selected samples with varying platelet contamination (P01-P05, P10). Data points indicate protein group identifications from three biological replicates. Bar heights represent the mean values, and the error bars present standard error of the mean.

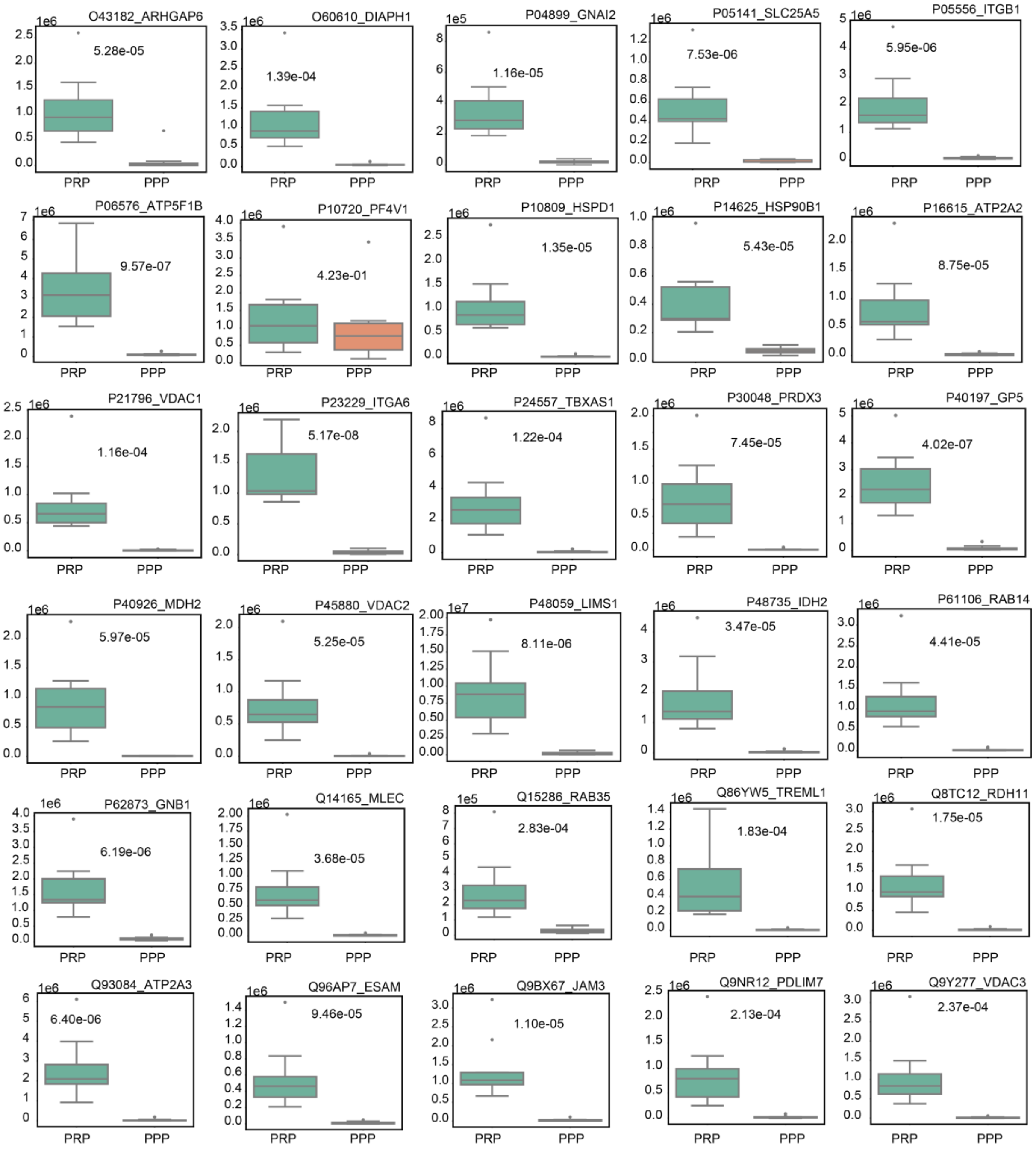

Appendix Figure S9 - **Comparative analysis of protein abundance distributions in matched PRP and PPP samples.**

The p values were assessed by paired Student's t-test for comparisons.

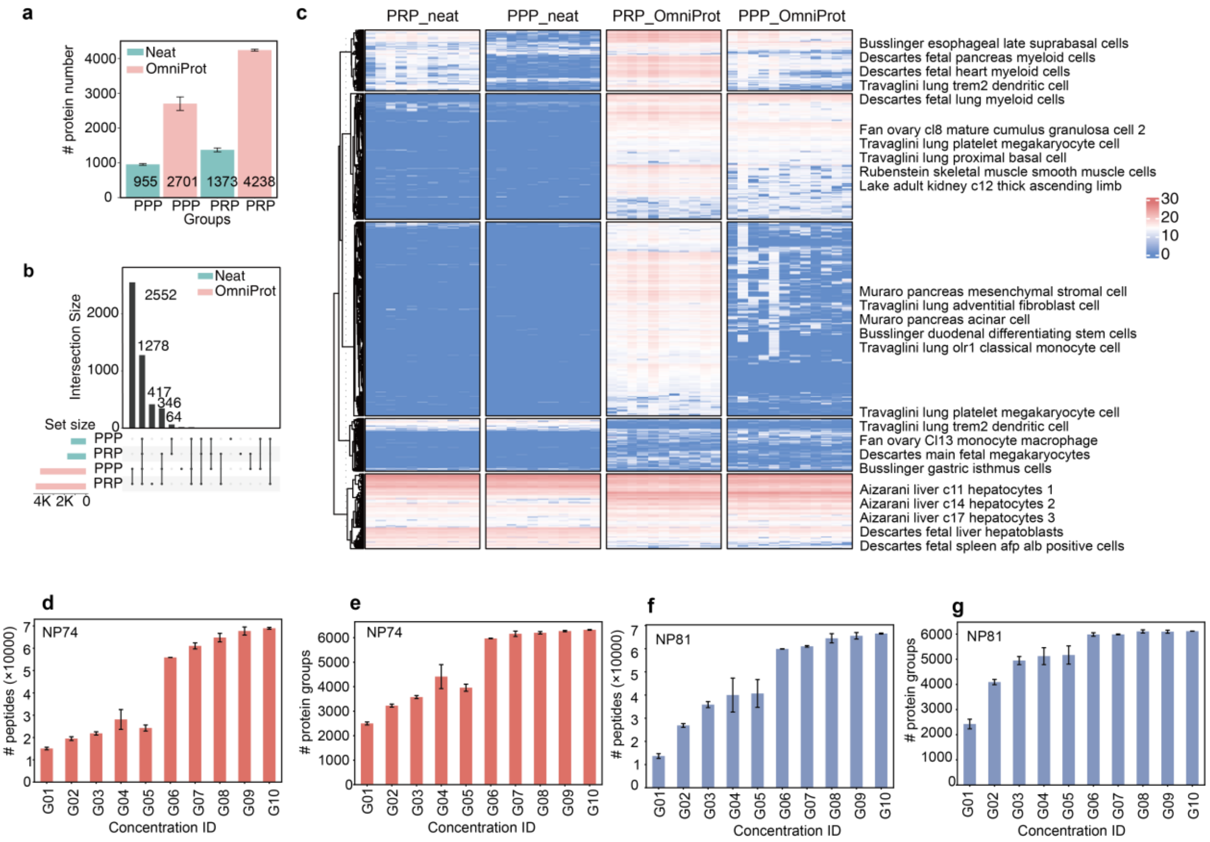

Appendix Figure S10 - **Heatmap of protein identification in 11 paired PRP and PPP samples**. (a) The number of proteins identified in 11 PRP and PPP patient samples under neat and OmniProt methods. (b) Upset plot comparing protein identification distribution in 11 PRP and PPP patient samples under neat and OmniProt methods. (c) The heatmap of protein identification in 11 paired PPP and PRP samples. The number of identified peptide precursors (d) and protein groups (e) in NP74. The number of identified peptide precursors (f) and protein groups (g) in NP81. Data points indicate protein group identifications from three biological replicates. Bar heights represent the mean values, and the error bars represent standard error of the mean.

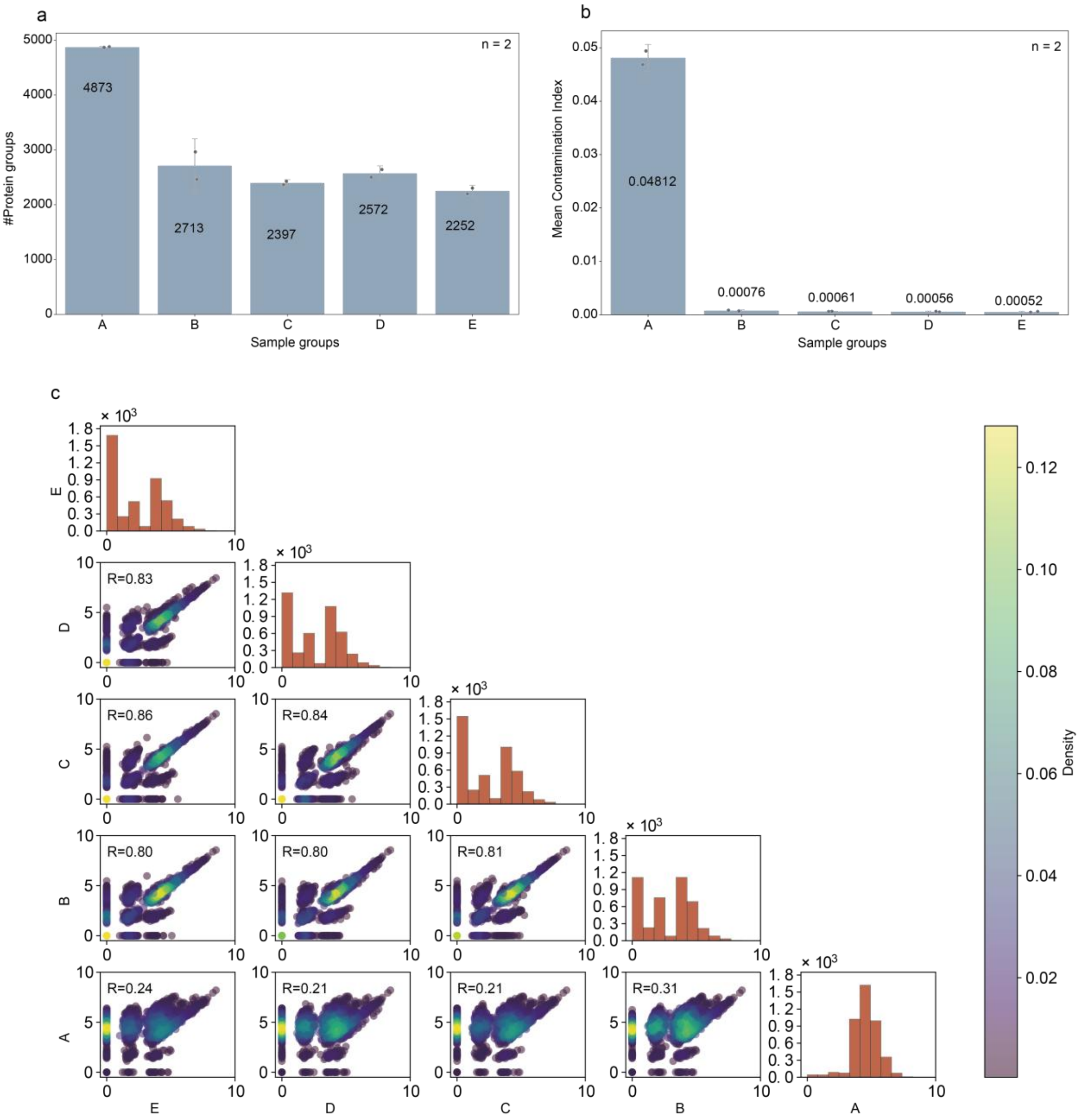

Appendix Figure S11 - **Comparison of platelet-rich plasma (PRP) samples and platelet-poor plasma (PPP) samples obtained using various centrifugation methods.**

(a) Comparison of the number of identified proteins across different types of plasma samples. (b) Comparison of platelet contamination index across different types of plasma samples. (c) Spearman correlation analysis of protein abundance across different plasma samples. Sample A represents PRP obtained by centrifugation at 200g for 15 minutes, while platelet-poor plasma samples were prepared using different centrifugation protocols including single centrifugation at 2000g for 15 minutes (sample B), double centrifugation at 2000g for 15 minutes (sample C), single centrifugation at 4000g for 15 minutes (sample D), and single centrifugation at 3000g for 30 minutes (sample E). Data points indicate protein group identifications from three biological replicates. Bar heights represent the mean values, and the error bars present standard error of the mean.

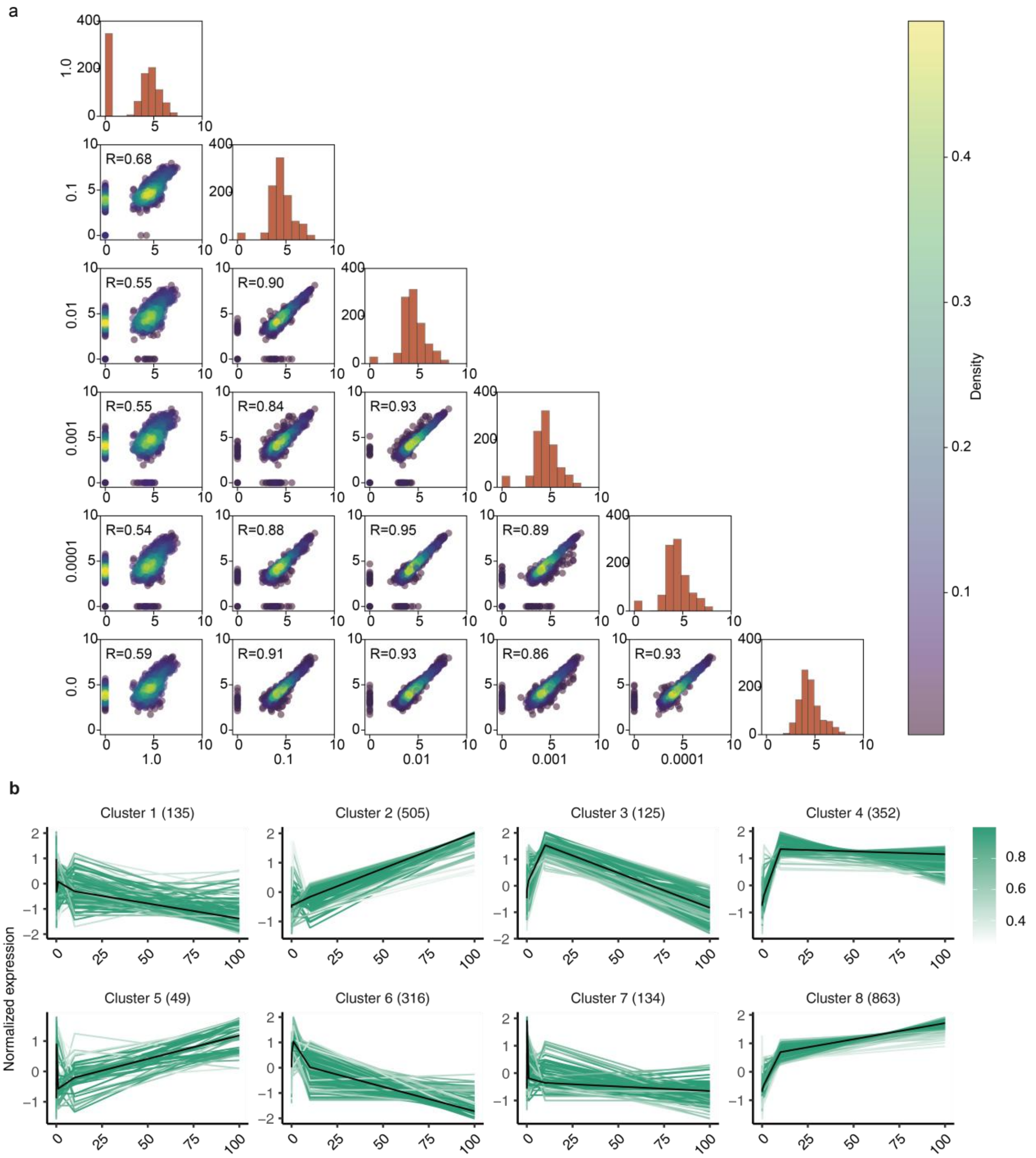

Appendix Figure S12 - **Erythrocyte contamination effects on OmniProt plasma proteome analysis.**

(a) Pearson correlation analysis of plasma proteome integrity across erythrocyte contamination gradients. (b) Mfuzz clustering analysis of proteins with <50% missing values.

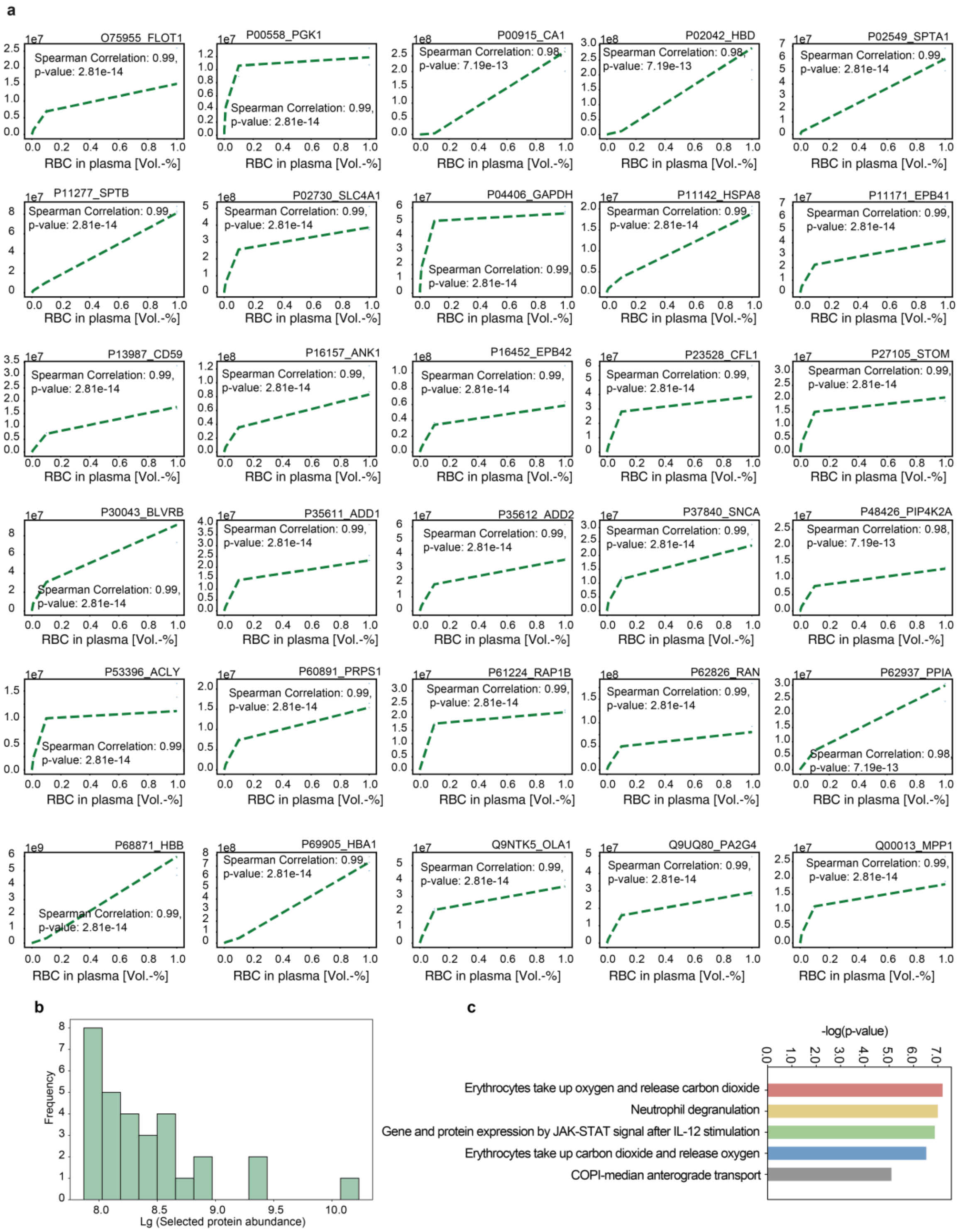

Appendix Figure S13 - **Correlation analysis of erythrocyte markers in OmniProt plasma samples.**

(a) The abundance distribution of selected 30 erythrocyte-related markers. (b) The abundance profiles of erythrocyte-associated markers identified in this study. (c) Pathway enrichment analysis of the 30 erythrocyte markers.

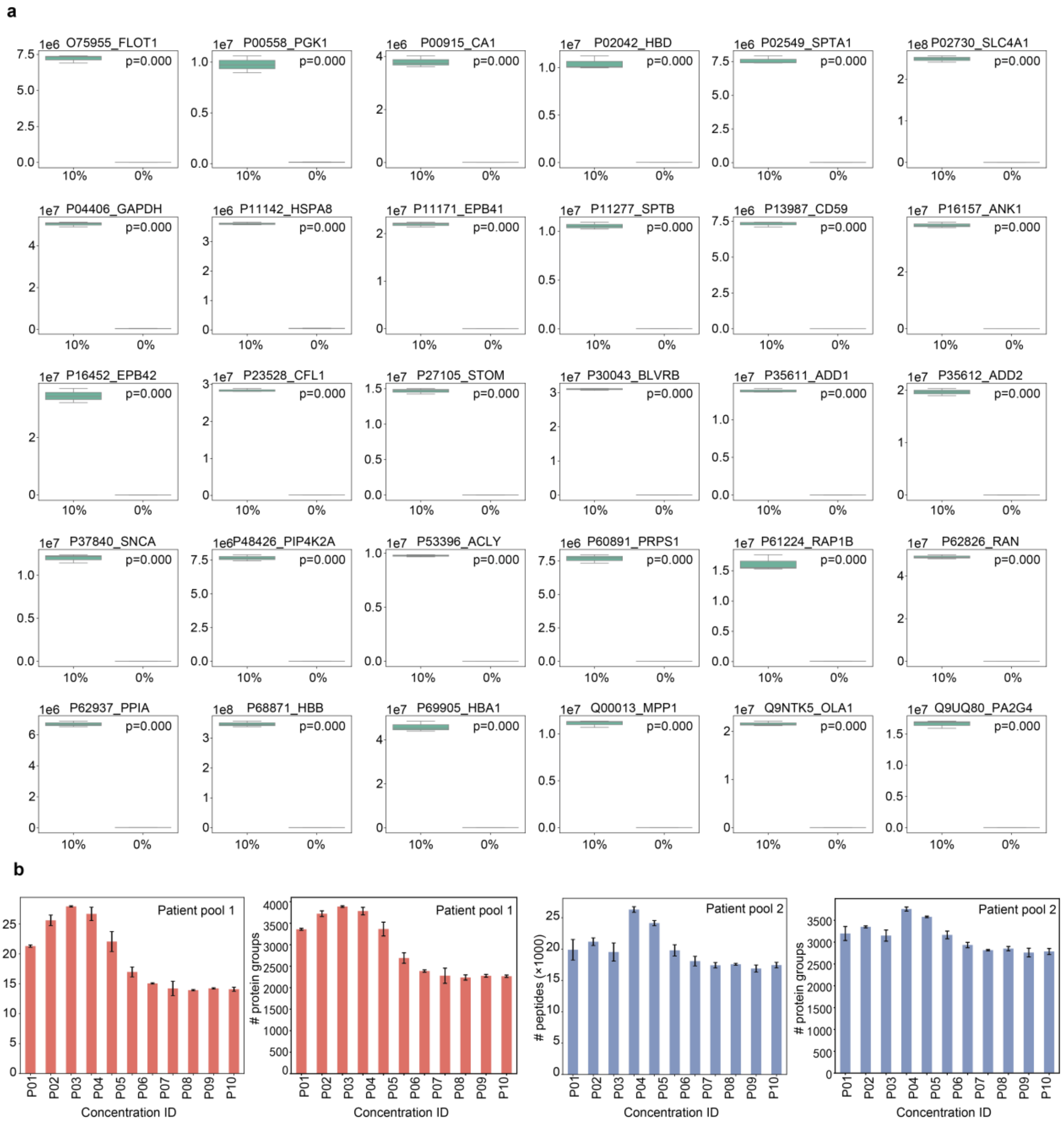

Appendix Figure S14 - **Validation of 30 erythrocyte markers in contaminated plasma samples**. (a) Protein abundance distribution comparison between 10% and 0% erythrocyte contamination samples. (b) Peptide precursor and protein group identifications across two patient pools (three patients per pool). P01-P10 represent serial dilutions of erythrocyte-contaminated samples with PPP, where P01 has the highest and P10 has the lowest level of erythrocyte contamination (dilution ratios shown in **Figure 5A**). Statistical significance was assessed by paired Student's t-test. Bar heights represent the mean values, and the error bars represent the standard error of the mean.
